# All-metallic high-efficiency generalized Pancharatnam–Berry phase metasurface with chiral meta-atoms

**DOI:** 10.1515/nanoph-2021-0811

**Published:** 2022-02-24

**Authors:** Jixiang Cai, Fei Zhang, Mingbo Pu, Yan Chen, Yinghui Guo, Ting Xie, Xingdong Feng, Xiaoliang Ma, Xiong Li, Honglin Yu, Xiangang Luo

**Affiliations:** State Key Laboratory of Optical Technologies on Nano-Fabrication and Micro-Engineering, Institute of Optics and Electronics, Chinese Academy of Sciences, Chengdu, 610209, China; Key Laboratory of Opto-electronic Technology and Systems of the Education Ministry of China, Chongqing University, Chongqing, 400044, China; School of Optoelectronics, University of Chinese Academy of Sciences Beijing, 100049, China

**Keywords:** all-metallic, chiral meta-atoms, generalized Pancharatnam–Berry phase, high-efficiency, metasurface

## Abstract

Metasurfaces based on the Pancharatnam–Berry (PB) phase have attracted significant attention in the domains of subwavelength optics and electromagnetics. Conventional theory predicts that the PB phase is exactly twice the rotation angle of the anisotropic meta-atoms. Differently, a recent advance has demonstrated that the generalized PB phase representing multiple times of the rotation angle could be obtained with high-fold rotational symmetry meta-atoms, but it suffers from the low cross-polarization conversion efficiency (the theoretical upper limit of 25%) that impedes its further applications, especially for meta-atoms with rotational symmetry ≥3. Here, we verify that the chiral meta-atoms with high-fold rotational symmetries could produce the generalized PB phase. Besides, the all-metallic configuration is utilized to design C2, C3, and C5 chiral catenary meta-atoms to improve their efficiency and bandwidth. The equivalent air waveguide with low loss between two adjacent meta-atoms is formed to analyse the higher performances of the all-metallic scheme for the realization of the generalized PB phase compared with the metal–insulator–metal and all-dielectric C3 meta-atoms. As a proof of concept, four metadevices including two spin-Hall metadevices and two holograms are experimentally demonstrated and their maximum efficiency could exceed 83% in simulation. This work could provide a high-efficiency platform for the study of the generalized PB phase in linear and nonlinear optics.

## 1 Introduction

Metasurfaces, comprising subwavelength artificial structures, enable flexible manipulation of the phase, polarization, and amplitude of light in reflection or transmission mode owing to its extraordinary electromagnetic characteristics [1]. By judiciously designing the dimension, rotation, and position of each unit element with high spatial resolution, the additional phase gradient or surface momentum can be generated for wavefront engineering of light at will, which promotes the evolution of Snell’s law to generalized Snell’s law [2], [3], [4]. This capability makes it extensively applied in holography [5], [6], [7], [8], planar lens [9], [10], [11], vectorial optical fields control [12], [13], [14], information encoding [15, 16], and so forth [17, 18]. In most circumstances, the wavefront manipulations of these functional metadevices rely on the propagation phase [19], geometric phase [20], detour phase [21], resonance phase [22], and others [23], [24], [25].

The PB phase derives from the coupling between intrinsic angular momentum and rotations of coordinates in inhomogeneous and anisotropic media, which can be obtained by locally rotating elements [2, 26, 27]. Under the illumination of circularly polarized light, the transmitted/reflected cross-polarized light will obtain a phase delay of Φ that is approximately linearly proportional to the orientation angle *θ* of the C2 nanostructures, i.e., Φ = 2*σθ*, where *σ* = ±1 indicates the left- and right-handed circular polarization (LCP and RCP), respectively [28], [29], [30]. However, it is commonly regarded to be isotropic and no geometric phase response for meta-atoms with the rotational symmetry ≥3 [31]. Therefore, a more general form of geometric phase is emerged in nonlinear metasurfaces at foremost, in which the phase factors are expressed as (*n* − 1)*σθ* and (*n* + 1)*σθ* (*n* is the order of harmonic generation) for nonlinear harmonic waves with the same or opposite helicity under the incidence of the circularly polarized fundamental wave, respectively [14, 32, 33]. Until recently, the principle of the generalized PB phase has been proposed in the linear optics regime with high-fold rotational symmetry meta-atoms, but the conversion efficiency of this monolayer plasmonic metasurface is restricted by the theoretically predicted upper limit of 25%, which hinders its further applications [34, 35]. Furthermore, the chiral meta-atoms are extensively applied in polarization conversion [36], optical chirality [37], and microwave absorption [38], in which few/multi-layer schemes are commonly utilized to improve its efficiency [39, 40]. Nevertheless, such a way not only increases the fabrication burden, but also may be difficult to realize the high-efficiency generalized PB phase limited by the interactions of different layers.

Here, we verify that the chiral meta-atoms could produce the generalized PB phase that is expressed as multiple times the rotation angle of the element in the linear optics. Moreover, the designed all-metallic catenary meta-atoms with high-fold rotational symmetries show high-efficiency and broadband wavefront manipulation capabilities compared to previous metal–insulator–metal (MIM) and all-dielectric configurations. As a proof of concept, several metadevices comprising the all-metallic C3 and C5 chiral meta-atoms are fabricated and experimentally demonstrated in the infrared regime, in which the measured efficiency could reach ∼59.1% (the simulated efficiency exceeds ∼80%). By virtue of its high-efficiency wavefront manipulation ability and large fabrication error tolerance, these finds could offer a platform for versatile applications, such as multifunctional light field modulation and tailoring linear and nonlinear optical responses.

## 2 Results and discussion

### 2.1 The principle and meta-atoms design

**Figure 1:**
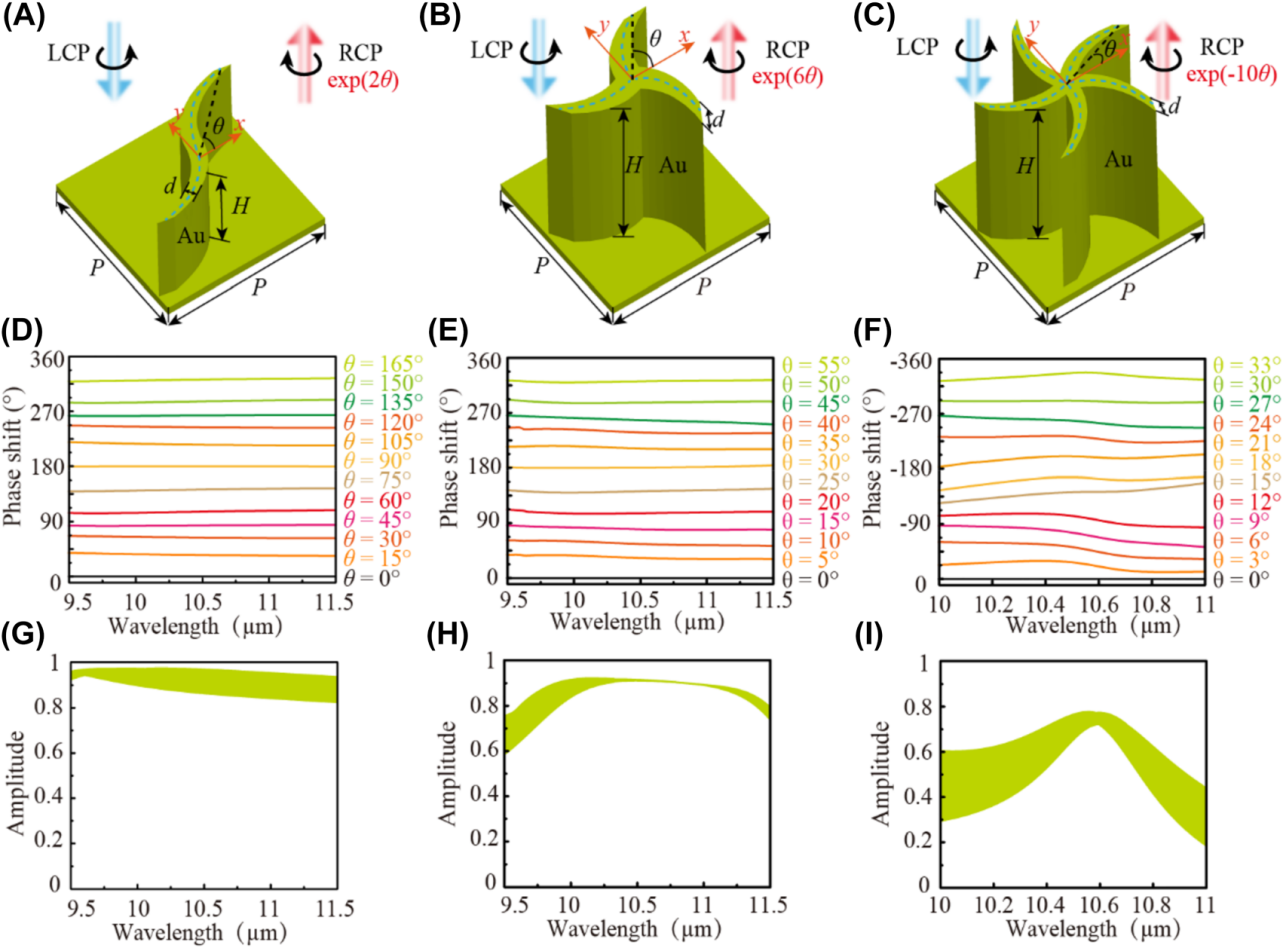
Numerically simulated results of the meta-atoms with different fold rotational symmetries. Schematic diagrams of (A) C2 meta-atom with *a* = 0.335, *d* = 1 μm, and *H* = 4 μm, (B) C3 meta-atom with *a* = 0.35, *d* = 1.5 μm, and *H* = 8 μm, and (C) C5 meta-atom with *a* = 0.33, *d* = 0.85 μm and *H* = 8 μm. These meta-atoms have the same lattice constant *P* = 8 μm. (D)–(F) The simulated phase shifts and (G)–(I) amplitude spectra of the reflected cross-polarized light as a function of orientation angle *θ* of ((D) and (G)) C2, ((E) and (H)) C3 and ((F) and (I)) C5 meta-atoms for LCP illumination.

The generalized PB phase derives from the lattice coupling effect, induced by an *n*-fold rotational symmetry meta-atom in the square lattice, could be expressed as [34]

(1) $$\Phi =\left\{ \begin{array}{l} \pm 2n\theta ,n\text{ is odd,}\\ \pm n\theta ,n\text{ is even.} \end{array}\right.$$

where the sign ± is determined by numerically investigating the rotating direction of the principal axis. Moreover, the generalized PB phase of meta-atoms in the hexagonal lattice will get a more complex expression in Reference [34]. To confirm this principle with chiral meta-atoms, we design three types of gold (Au) catenary meta-atoms with C2, C3, and C5 rotational symmetries as shown in Figure 1A–C, respectively, because it facilitates the construction of planar chiral meta-atoms by rotating catenary structure. The material parameter of gold used in simulations is shown in Supplementary Figure S1. The blue dashed line (midline) that denotes the catenary curve can be described as [23]:

(2) $$y=\frac{\Lambda }{\pi }\ln\left(\left|\sec\left(\pi x/\Lambda \right)\right|\right)\;x\in \left(0,a\Lambda \right)$$

where Λ is the horizontal length of a single catenary, and *a* is the truncated coefficient. The range of *x* is set to be positive, which implies that only half of the catenary is exploited to construct the chiral meta-atoms. Note that the angle between two adjacent blue dashed lines is 180°, 120°, and 72° for C2, C3, and C5 meta-atoms, respectively.

According to Eq. (1), the chiral C2 meta-atom consisting of catenary arrays can generate the PB phase of ∼2*σθ*, whereas the C3 and C5 meta-atoms could obtain high-fold phases of ∼6*σθ* and ∼−10*σθ*, respectively. For the C5 meta-atom, the minus stems from the fact that the rotating direction of the principal axis is flipped compared with the C2 and C3 meta-atom [34], as shown in Supplementary Figure S2. To confirm the general PB phase and feature the high performance of meta-atoms, numerical simulations are performed using the finite element method (FEM) in a commercial software package CST Microwave Studio. As shown in Figure 1D–F, the phase shifts for the C2, C3, and C5 chiral meta-atoms can cover the whole 2*π* range when the orientation angles *θ* change 180°, 60°, and 36°, respectively, which are agree well with theoretical expectations. In addition, phase shifts are not exact multiple times of *θ* due to the influence of the spin-independent propagation or resonance phase [34]. The amplitudes of the reflected cross-polarized light of C2, C3, and C5 meta-atoms with different orientations are illustrated in Figure 1G–I, which exceed ∼0.89, ∼0.91, and ∼0.75 at the central wavelength of 10.6 μm (the working wavelength of the CO_2_ laser), respectively. These performances are affected by the dimensions of meta-atoms, in which the bandwidth can be increased/reduced as the period reduces/increases. Furthermore, the red/blue shift can be observed in phase curves as the period of meta-atom increases/reduces. Its influence on amplitude and phase is small with the period varying from 7.7 μm to 8.3 μm (more dimensions effects of period *P*, height *H*, distance *d*, and coefficient *a* are shown in Supplementary Figure S3). Especially, the amplitude (*θ* = 0°) is ∼0.9 for chiral C2 meta-atom in a broad wavelength range from 9.5 to 11.5 μm. Notably, all the designed chiral meta-atoms possess the almost same amplitudes for LCP and RCP illumination.

### 2.2 Comparison of all-metallic meta-atom and other structures

**Figure 2:**
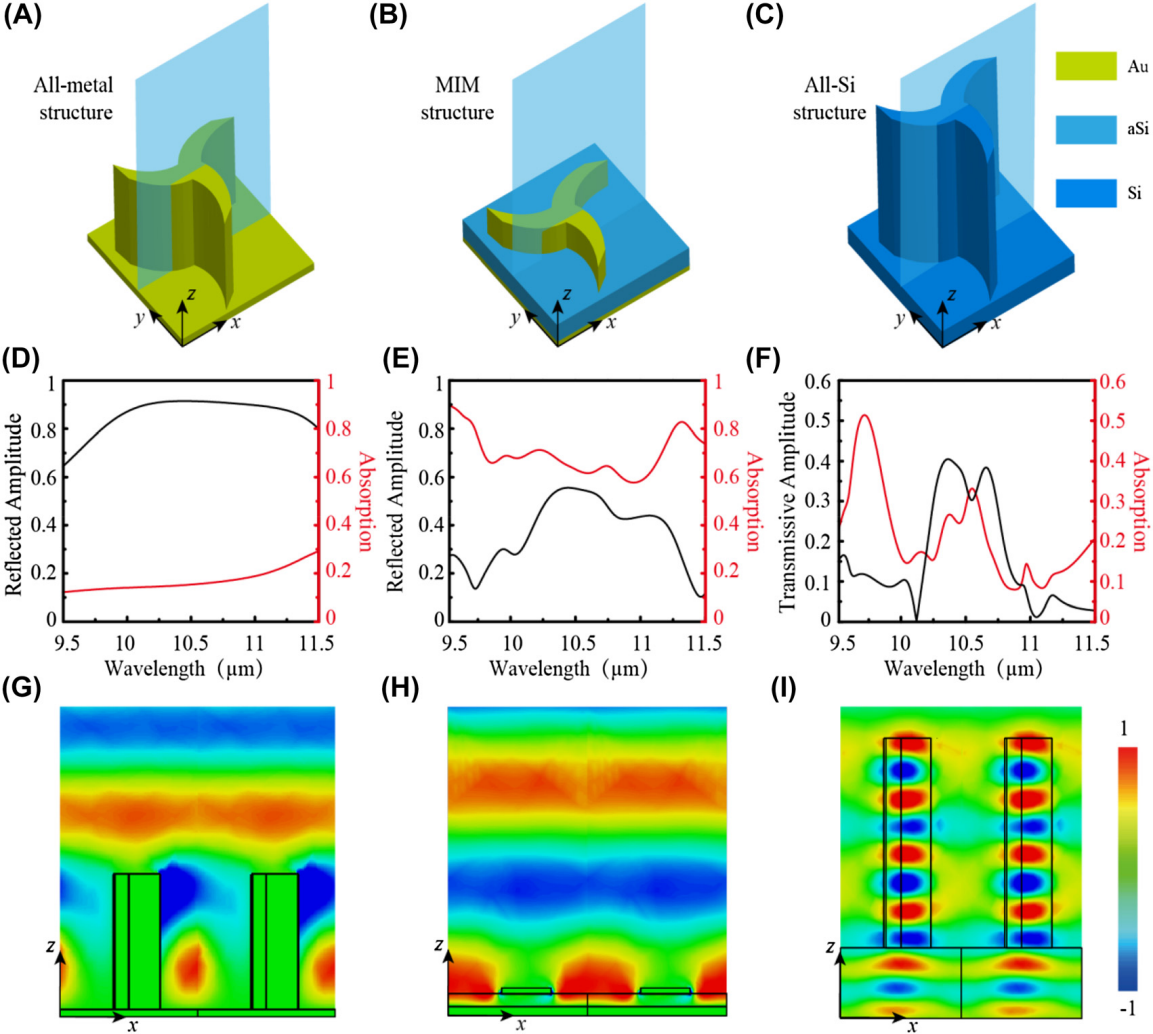
Numerically simulated results of the all-metallic, MIM, and all-dielectric C3 meta-atoms. (A)–(C) Schematic diagrams of the all-metallic, MIM (Au–aSi–Au, aSi is amorphous silicon), and all-dielectric (Si) C3 meta-atoms. The dimension of MIM meta-atom is *a* = 0.325, *d* = 1.55 μm, *H* = 0.3 μm (the thickness of the top layer Au) and the thickness of aSi is 0.7 μm. For all-dielectric meta-atom, *a* = 0.3, *d* = 1.8 μm. The refractive index of the materials is from Reference [46]. (D)–(F) Amplitude spectra of the cross-polarized light and absorption spectra for three meta-atoms. (G)–(I) The distributions of electric field **E**_*x*_ along the *xoz* (*y* = 0) plane between two adjacent meta-atoms for LCP illumination.

In order to show the advantages of the all-metallic metasurface, we numerically simulate the MIM and all-dielectric meta-atoms as a comparison with the all-metallic configuration. All the meta-atoms are optimized to obtain the maximum efficiency and bandwidth with the same lattice constant (*P*) but diverse sizes and their schematic diagrams are illustrated in Figure 2A–C. Corresponding simulated results are plotted in Figure 2D–F, in which the all-metallic C3 chiral meta-atom could obtain a higher average amplitude of ∼0.86 in the range of 9.5–11.5 μm as illustrated in Figure 2D. Meanwhile, its average absorption is lower than ∼16%. For the MIM meta-atom in Figure 2E, the amplitude is much smaller than the all-metallic one about 0.37 in this broad range, which reaches the maximum of ∼0.56 at 10.4 μm. This is restricted by large absorption ∼70% on average. However, the all-dielectric meta-atom (in Figure 2F) suffers from a lower average transmissive amplitude of ∼0.36 (the maximum is ∼0.4 at 10.36 μm) and a narrower bandwidth of 10.25–10.75 μm, simultaneously. It also reveals that the average absorption is ∼24%, and thus the most incident energy (∼46%) is straightforwardly reflected.

To get a clear visualization of the abovementioned schemes, the instantaneous electric field distributions **E**_*x*_ in the *xoz* plane for LCP incidence at the resonant wavelength of 10.6 μm are shown in Figure 2G–I. The metal material (in the near- and mid-infrared range could be recognized as the perfect electrical conductor with neglected metal loss resulting in strong reflection of incident light near 100% (Supplementary Figure S1). As illustrated in Figure 2G, when the electromagnetic wave goes through the meta-atom, the low loss air waveguide is produced to generate high polarization conversion efficiency. Here, its physical mechanism is modeled by the catenary theory, in which the effective impedance of the metal array is described by the catenary function [41]. Limited by the substantial ohmic loss induced by the Fabry–Perot effect of the conventional MIM configuration with C2 nanobricks, its efficiency is hard to reach 80% [20, 41]. Especially, for *n*-fold rotational symmetry meta-atoms (in Figure 2H), the optical anisotropy is weakened along with *n* increasing, hence the thickness of the dielectric layer should be properly increased to reduce this effect. Unfortunately, such a way may induce strong field confinement and lead to larger absorption. Besides, the action of hybridmodes, surface plasmons mode, and near field interaction between adjacent elements also may enhance absorption [42, 43]. Thus, the efficiency is more difficult to reach 80% for multi-fold rotational symmetry meta-atoms. An alternative approach is employing the high aspect ratio all-dielectric meta-atom that can be considered as the truncated waveguide as shown in Figure 2I [10, 44]. Similar to the MIM meta-atom, the optical anisotropy for high-fold rotational symmetry meta-atoms could be enhanced by increasing the height (here, the height of Si meta-atom is 15 μm, larger than the wavelength of incident light), but it increases the fabrication burden and may reduce its working bandwidth. Besides, it may induce lower transmissive conversion efficiency due to the large refractive index of the substrate of meta-atom leading to large reflection [45]. Although the efficiency could be increased by utilizing a substrate with the low refractive index, it also increases fabrication complexity and cost, especially for high-fold rotational symmetries meta-atoms. In general, the all-metallic method exhibits higher performance for realizing the high-efficiency and broadband generalized PB phase compared with MIM and all-dielectric schemes.

## 3 Experiments and characterization

**Figure 3:**
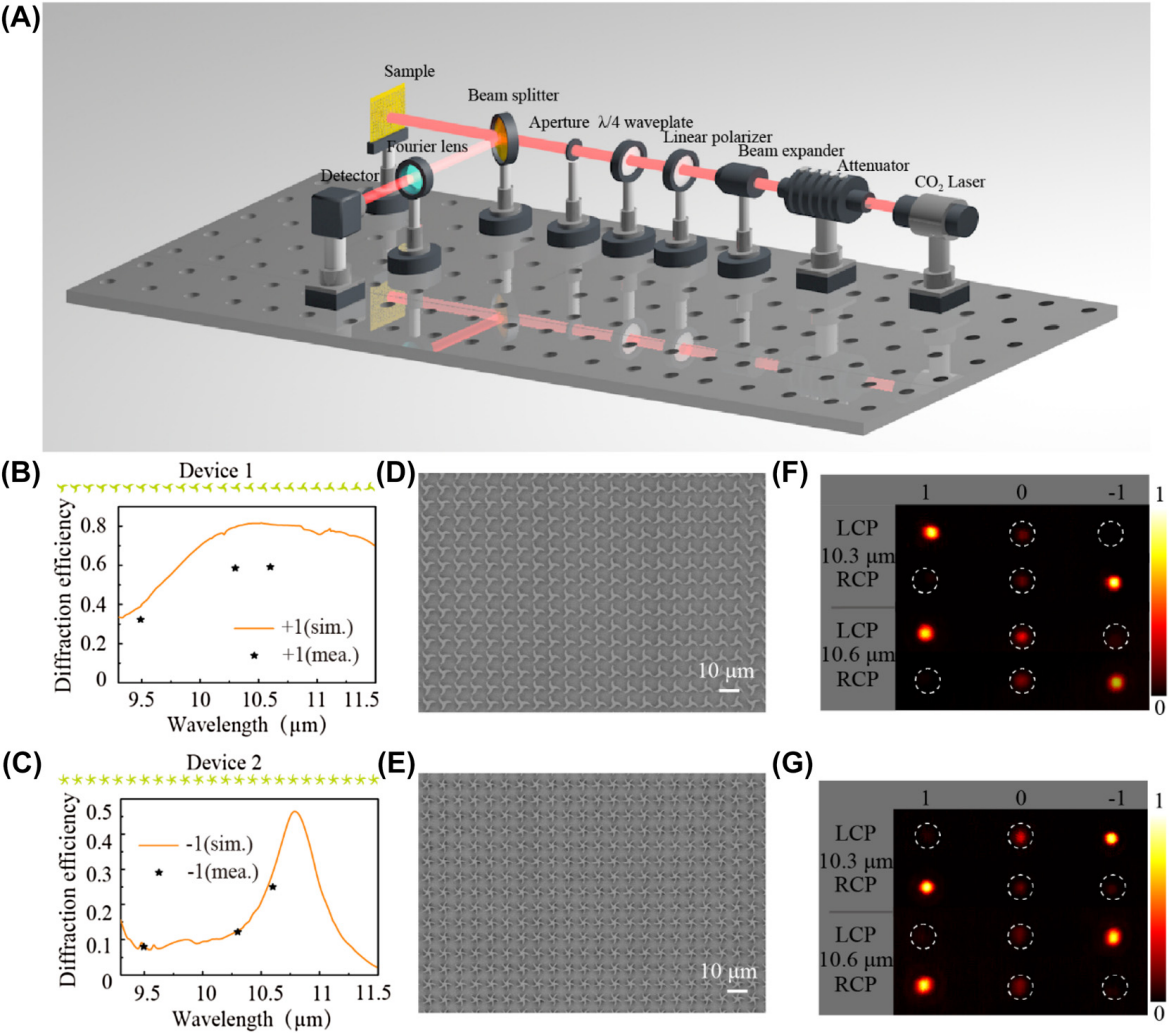
Optical setup and experimental results of the spin-Hall metadevices. (A) Schematic illustration of the measurement optical setup. (B) and (C) Simulated (orange lines) and measured (black stars) efficiencies of two fabricated metadevices for LCP illumination. (D)–(E) SEM images of two devices with the size of 0.5 × 0.5 cm^2^. (F) and (G) Measured reflected patterns of two devices at the wavelength of 10.3 and 10.6 μm under the illumination of LCP and RCP. The constant background produced by thermal radiation has been removed.

To experimentally verify the high-efficiency and broadband phase modulation capabilities of multi-fold (≥3) rotational symmetry meta-atoms, four metadevices (two spin-Hall metadevices and two holograms), composed of spatially varying C3, and C5 chiral meta-atoms, are fabricated as follows. First, a 1 μm thick positive photoresist (Pr) AZ1500 is spin-coated (2200 rpm) onto the cleaned silicon (Si) wafer and baked at 100 °C for 10 min. Then, the direct writing lithography is used to transfer the patterns of elements into the Pr layer. Next, the Pr pattern is transferred to the Si wafer by inductively coupled plasma etching, and then the Pr layer is removed by oxygen (O_2_). Finally, a 300 nm thick gold layer (larger than skin depth) is deposited on the Si wafer through magnetron sputtering, and thus it can be recognized as an all-metallic one. For the performance characterization of the fabricated metadevices, the experiment measurement optical setup is shown in Figure 3A. A CO_2_ laser is utilized as the light source. The optical beam successively passes through an adjustable attenuator, a linear polarizer, a quarter waveplate, an adjustable aperture, and then illuminates the sample from the top of the metadevices. A beam splitter is placed in front of the sample to guide the reflected diffraction patterns to the charge-coupled detector (384 × 288 pixels, UA330, Guide-Infrared Inc.) with the size of each pixel of 25 μm × 25 μm. For the efficiency characterization, the detector is replaced with an infrared power meter.

Two spin-Hall metadevices are designed by periodically arranging two arrays (inserts at the top of Figure 3B and C) of 24 meta-atoms with an interval of ∼*π*/12, respectively. This phase interval creates a small deflection angle that is convenient for the detection of the diffracted beam. Figure 3B and C likewise illustrate the simulated absolute efficiencies as a function of wavelength under LCP illumination. Here, the absolute efficiency is defined as the ratio of the power of one order to the total incident power [41]. Evidently, most of the power is reflected to the +1st order for device 1, but the −1st order for device 2 due to the rotation of the principal axis resulting from the lattice coupling effect. Corresponding efficiencies surpass ∼80%, and ∼29% at the central wavelength of 10.6 μm, respectively. In addition, the average efficiency of device 1 is up to ∼83% in a broad range of 9.5–11.5 μm. Figure 3D–G illustrates the scanning electron microscope (SEM) images and their measured diffraction patterns of two devices. The measured +1st and −1st order bright spots are caused by the anomalous reflection of two converted spin components, while the central dim spot (0th order) stems from the unmodulated spin component of the reflected beam. The measured efficiencies of the two devices exceed ∼58.5%, and ∼12.2% at 10.3 μm for LCP illumination, while higher efficiencies reach ∼59.1%, and ∼24.7% at 10.6 μm, respectively. To further demonstrate the broadband characteristic of the designed metadevices, we also measure the efficiencies of ∼32.4% and ∼7.9% at 9.3 μm for two metadevices. As a comparison with simulated results, the measured results are added in Figure 3B and C (black stars), in which the selected wavelength is decided by our laser. The difference between simulation and measurement results can be observed, which is attributed to the imperfection of fabrication and measurement errors (such as the effect of geometrical parameters in Supplementary Figure S3 and inhomogeneous thickness of the gold layer on the top and sidewalls in Supplementary Figure S4).

**Figure 4:**
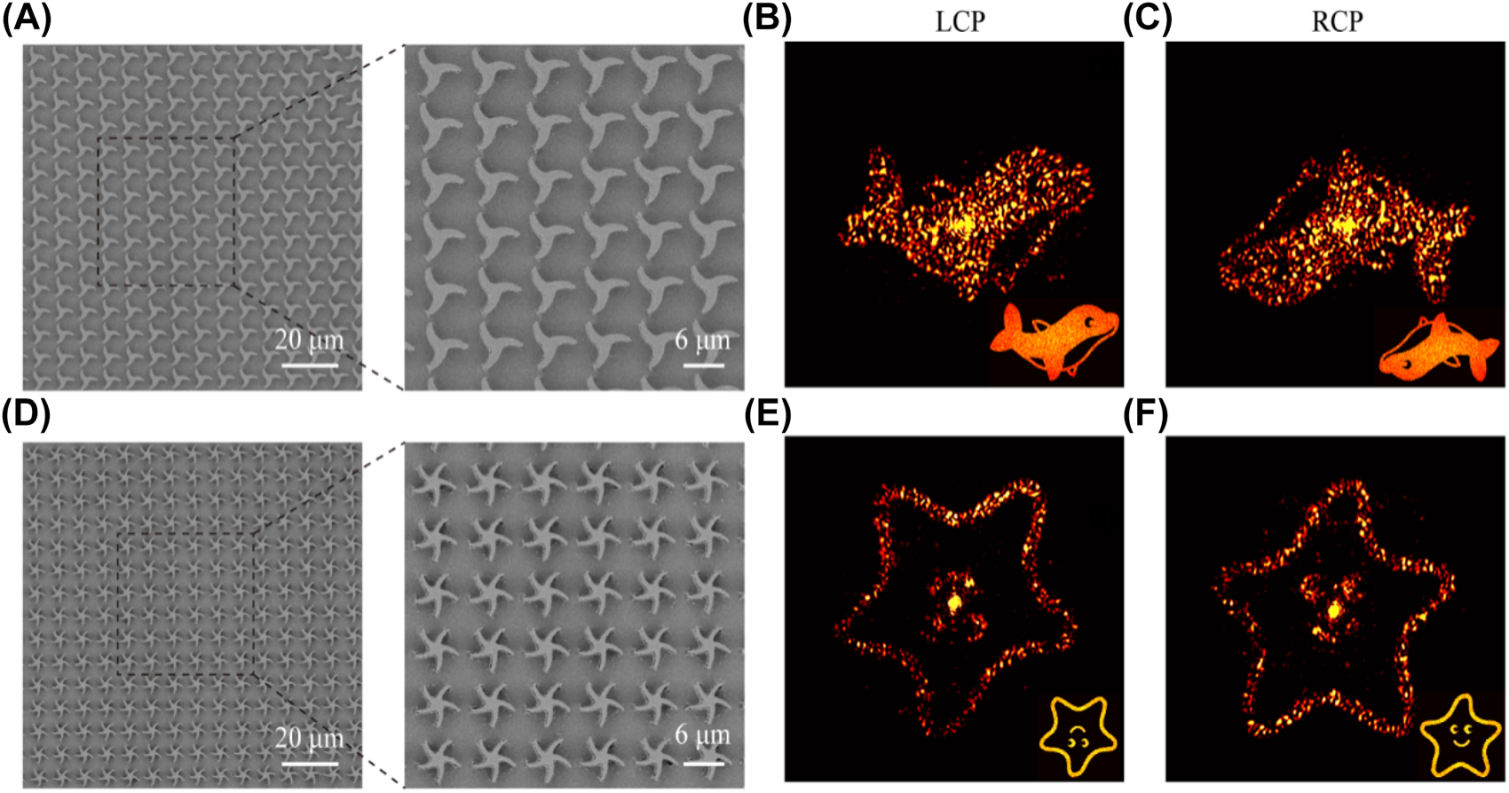
Experimental demonstrations of holographic samples. (A) and (D) SEM images of two metahologram samples. (B), (C), (E) and (F) Measured and theoretical (in the lower right corner) diffraction patterns produced by two metaholograms under LCP (left) and RCP (right) illumination. The constant background produced by thermal radiation has been removed.

Besides, two phase-only holographic samples (the area of 1 × 1 cm^2^) are fabricated to further demonstrate the high performances as shown in Figure 4. Note that a beam expander in Figure 3A is inserted into the experimental setup to measure the holographic images. Each phase map of the holographic images is computed based on the Gerchberg–Saxton algorithm. According to the relation of Φ–*θ*, the hologram can be coded by rotating the chiral C3 catenary meta-atoms from 0–60°, and its SEM images are shown in Figure 4A. Two centrosymmetric diffraction patterns of cartoon dolphins are measured in Figure 4B and C. This phenomenon is caused by the sign of the phase profile flips along with the incident light changing from LCP to RCP [47, 48]. By arranging the chiral C5 meta-atoms from 0 to 36°, another hologram is obtained and its SEM images are shown in Figure 4D (the SEM images of the sample with a tilt angle are shown in Supplementary figure S4). Although two centrosymmetric patterns, cartoon starfish, can be reconstructed in Figure 4E and F, the images are swapped for LCP and RCP illumination unlike the abovementioned results. This is due to the influence of the rotating direction of the principal axis. The measured results are in good agreement with theoretical designs, which demonstrates the great performances of the all-metallic configuration.

## 4 Conclusions

In summary, we present the all-metallic metasurfaces to realize the high-efficiency generalized PB phase by chiral meta-atoms with high-fold rotational symmetries in the reflection mode. The efficiency of this methodology could surpass ∼80%, which is much higher than the theoretically predicted upper limit of ∼25% of single-layer plasmonic metasurfaces. By locally controlling the symmetry and phase profile, several spin-Hall and holographic metadevices are designed, fabricated, and demonstrated experimentally, whose performances agree well with numerical simulations. Benefiting from the high strength, high electric conductivity, and large error tolerance of the metal materials, this all-metallic scheme is easy to realize large-area fabrication combining electroplating possess and nanoimprint and may find practical applications in linear and nonlinear optics for vector optical field control and high-efficiency planar devices like reflective diffractive lightsails [49, 50].

## Supplementary Material

Supplementary Material Details
