# Supplementary material for: All-metallic high-efficiency generalized Pancharatnam–Berry phase metasurface with chiral meta-atoms
Source: Nanophotonics. 2022 Feb 24;11(9):1961–8. doi: 10.1515/nanoph-2021-0811 (PMC11501248; doi:10.1515/nanoph-2021-0811)
Supplement: Supplementary file 1 — Supplementary Material Details [file j_nanoph-2021-0811_suppl.docx]

All-metallic high-efficiency generalized Pancharatnam- Berry phase metasurface with chiral meta-atoms

Jixiang Cai^a^, Fei Zhang^a^, Mingbo Pu, Yan Chen, Yinghui Guo, Ting Xie, Xingdong Feng, Xiaoliang Ma, Xiong Li, Honglin Yu, and Xiangang Luo*

**^a^Jixiang Cai and Fei Zhang:** These authors contributed equally to this work.

***Corresponding author: Xiangang Luo,** State Key Laboratory of Optical Technologies on Nano-Fabrication and Micro-Engineering, Institute of Optics and Electronics, Chinese Academy of Sciences, Chengdu 610209, China; and School of Optoelectronics, University of Chinese Academy of Sciences, Beijing 100049, China, e-mail: lxg@ioe.ac.cn. https://orcid.org/0000-0002-1401-1670.

**Jixiang Cai, Fei Zhang, Mingbo Pu, Yan Chen, Yinghui Guo, Ting Xie, Xingdong Feng, Xiaoliang Ma and Xiong Li,** State Key Laboratory of Optical Technologies on Nano-Fabrication and Micro-Engineering, Institute of Optics and Electronics, Chinese Academy of Sciences, Chengdu 610209, China.

**Jixiang Cai and Honglin Yu,** Key Laboratory of Opto-electronic Technology and Systems of the Education Ministry of China, Chongqing University, Chongqing 400044, China.

**Mingbo Pu, Yinghui Guo, Xiaoliang Ma and Xiong Li,** School of Optoelectronics University of Chinese Academy of Sciences Beijing 100049, China.

**S1. Simulations: The permittivity of the gold and its reflectance and absorption distribution.**

The permittivity of the gold (Au) and its reflectance and absorption distribution, only considering the intraband absorption in infrared range, is plotted in Figure S1(a) and Figure S1(b), respectively. The wavelength-dependent permittivity of Au could be described by:

$\varepsilon\text{=}\varepsilon_{1}+i\varepsilon_{2}$ (S1)

where ε_1_ and ε_2_ are the real part and imaginary part of the complex permittivity, respectively. By parametric fitting, the plasma frequency *w_p_* and collision frequency *w_τ_* are 1.1962×1016 rad s^-1^ and 1.125×1014 rad s^-1^, respectively.

The intraband absorption (main absorption of metal) can be approximated by [1]:

$A\approx\sqrt{\frac{2w}{\pi\sigma_{0}}}$ (S2)

where *σ_0_* is electrical direct current conductivity that is related to *w_p_* and *w_τ_* [2]. According to equation (S2), the absorption (A) and reflectance (1-*A*) of Au are depicted in Figure 1(c), whose general tendencies are consistent with previous works [3, 4]. Therefore, the Au material can be regarded as the perfect conductor, and almost all the incident power within the broadband spectrum range is directly reflected.

**
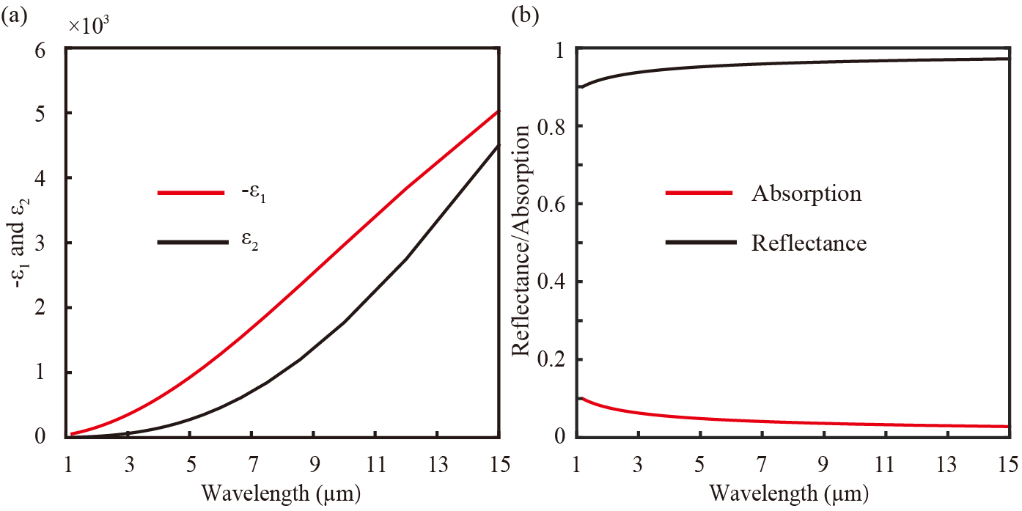
**

**Figure S1.** (a)The permittivity of the gold. (b) The reflectance and absorption distribution

**S2. Relation between the principal axes and the rotation angle of meta-atoms**

For the chiral C2 meta-atom, it can generate a geometric phase of ±2*θ* (in manuscript) on the reflected light with opposite handedness. However, it will generate phase shifts of 6*θ* (−10*θ*) for the C3 and C5 meta-atoms, in which the minus for the C5 meta-atom originates from the flipping of the rotation direction of the principal axis as shown in Figure S2. For higher-fold rotational symmetries meta-atoms, the investigation of the rotating direction of the principal axis is shown in ref [34].


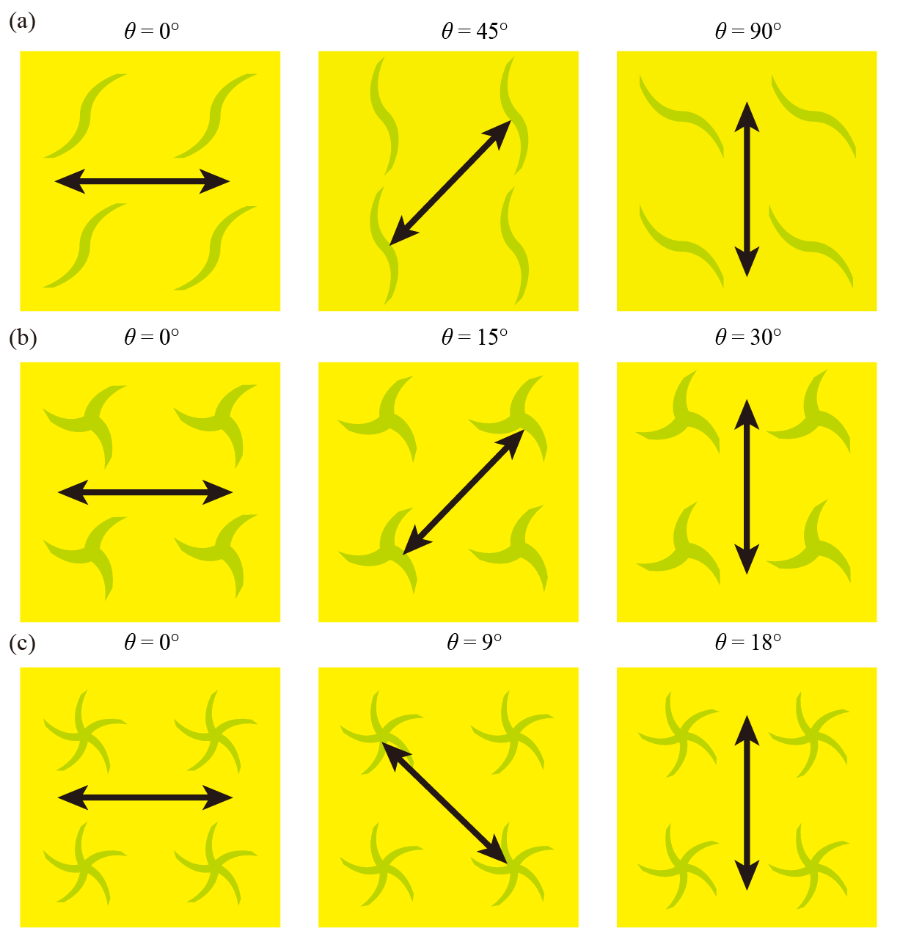


Figure S2. Rotation dependence of the principal axis and meta-atoms with (a) C2, (b) C3, and (c) C5 rotational symmetry. The double-headed arrows denote the orientations of the principal array axis, which correspond to the evolution of the polarization states.

**S3. Geometrical parameters and their effects**

**
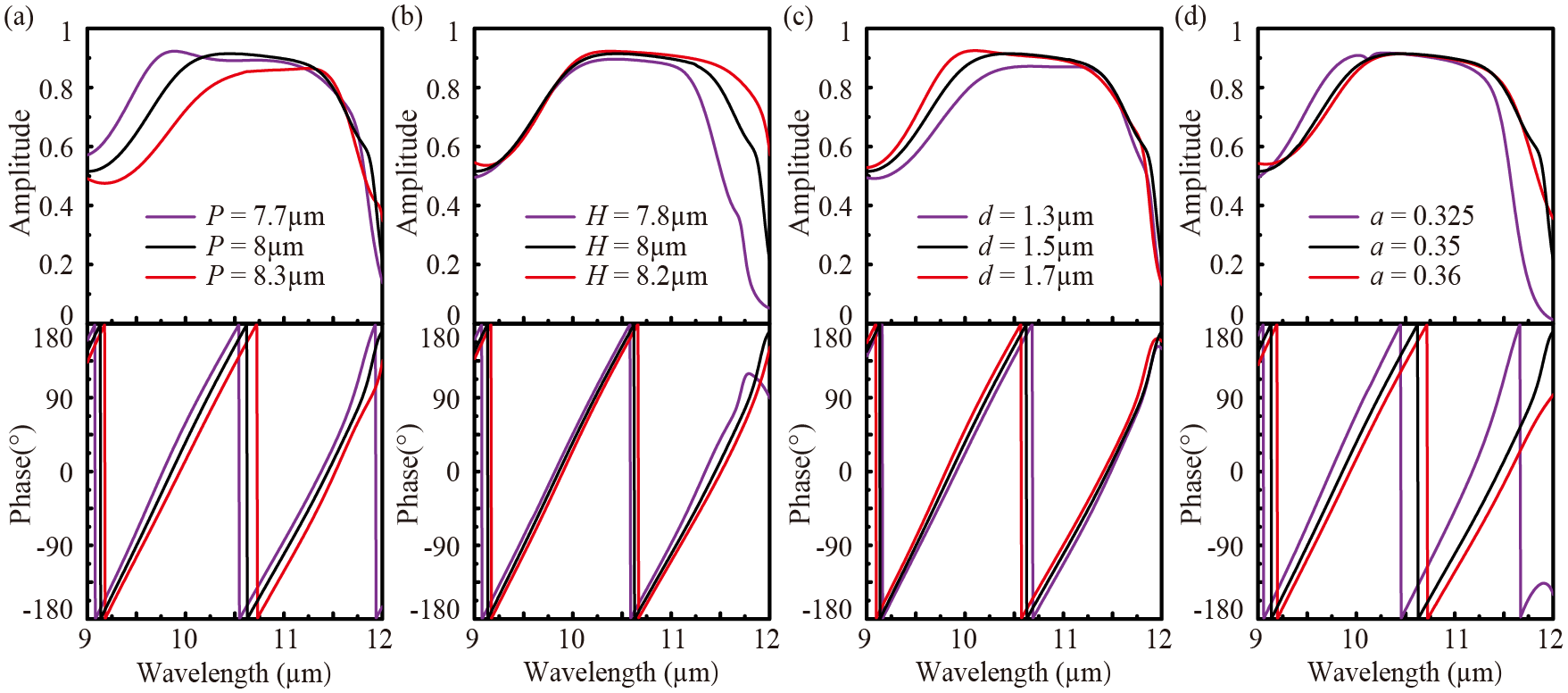
**

Figure S3. The effect of (a) period *P*, (b) height *H*, (c) distance *d*, and (d) coefficient *a* of C3 meta-atom under LCP incidence. The bandwidth can be increased/reduced as the *H* and *d* increase/reduce (*P* and *a* reduce/increase). The slight red/blue shift for phase spectra can be observed as the period *P*, height *H*, and coefficient *a* increase/reduce (*d* reduces/increases).

**S4. The scanning electron microscope images of the sample with a tilt angle**

Figure S3 shows the SEM images of the sample with a tilt angle for the C5 meta-atom. Due to the high aspect ratio of meta-atoms, the sidewall will be slightly tilted by inductively coupled plasma (ICP) etching. A 300 nm thick gold layer (larger than skin depth) is deposited on the Si wafer through magnetron sputtering. It has been demonstrated that the thickness of gold layers is not the same on the top and sidewalls [41]. Furthermore, the poor adhesion between the gold and Si layer also will reduce the performances of metadevices, which can be improved by adding a chromium layer between them.

**
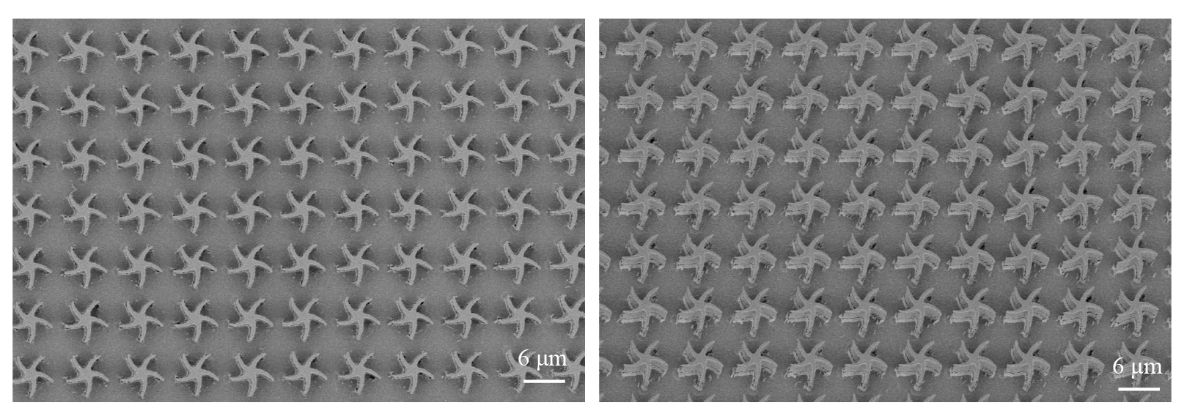
**

Figure S4. The SEM images of the sample with a tilt angle for the C5 meta-atom

**References**

[1] Bergström D, Kaplan A, and Powell J. Mathematical modelling of laser absorption mechanisms in metals: A review. The Absorption of Laser Light by Rough Metal Surfaces 2003;19.

[2] Ordal MA, Long LL, Bell RJ, et al. Optical-properties of the metals al, co, cu, au, fe, pb, ni, pd, pt, ag, ti, and w in the infrared and far infrared. Appl Opt 1983;22:1099-1119.

[3] Prokhorov AM, Laser heating of metals (CRC Press, 2018).

[4] Hagen E and Rubens H. ber beziehungen des reflexions- und emissionsvermögens der metalle zu ihrem elektrischen leitvermögen. Ann Phys-Berlin 1903;316:873-901.
